# RNAi-mediated treatment of two vertically transmitted rhabdovirus infecting the salmon louse (*Lepeophtheirus salmonis*)

**DOI:** 10.1038/s41598-017-14282-3

**Published:** 2017-10-25

**Authors:** Aina-Cathrine Øvergård, Lars Are Hamre, Heidi Kongshaug, Frank Nilsen

**Affiliations:** SLCR-Sea Lice Research Centre, Department of Biology, University of Bergen, Thormøhlensgt. 55, Pb. 7803, NO-5020 Bergen, Norway

## Abstract

Rhabdoviruses are a family of enveloped negative-sense single-stranded RNA viruses infecting a variety of hosts. Recently, two vertically transmitted salmon louse (*Lepeophtheirus salmonis*) rhabdoviruses (LsRV) have been identified. The prevalence of these viruses was measured along the Norwegian coast and found to be close to 100%, and with the present lack of suitable cell lines to propagate these viruses, it is challenging to obtain material to study their host impact and infection routes. Thus, virus free lice strains were established from virus infected lice carrying one or both LsRVs by treating them with N protein dsRNA twice during development. The viral replication of the N protein was specifically down-regulated following introduction of virus-specific dsRNA, and virus-free lice strains were maintained for several generations. A preliminary study on infection routes suggested that the LsRV-No9 is maternally transmitted, and that the virus transmits from males to females horizontally. The ability to produce virus free strains allows for further studies on transmission modes and how these viruses influences on the *L*.*salmonis* interaction with its salmonid host. Moreover, this study provides a general fundament for future studies on how vertically transmitted rhabdoviruses influence the biology of their arthropod hosts.

## Introduction

RNA interference (RNAi), sequence-specific post-transcriptional gene silencing, was initially discovered in transgenic tobacco plants expressing untranslated sense or antisense RNAs of a viral coat-protein gene^1,2^. The transgenic plants showed anti-viral activities, however, it was later that the requirement for double stranded RNA (dsRNA) to activate the RNAi pathway was reported^3^. Now it is clear that RNAi is a conserved mechanism in eukaryotic cells that can silence the gene expression of both viral and endogenous genes. Hence, it has been postulated to be a promising therapeutic method for viral diseases such as HIV, hepatitis B and the Ebola virus^4–6^.

As the major challenge regarding the use of RNAi as antiviral treatment in mammals has been the delivery method^7,8^, this has been more promising in invertebrates due to the systemic nature of RNAi in many of these species. Trials in farmed shrimps have shown that muscular injection of dsRNA can be used therapeutically against yellow head virus and infectious myonecrosis virus, at least to decrease the viral load and increase survival rate of infected animals^9–11^. In insects such as the honeybee, oral treatment with dsRNA simultaneously with virus inoculation has been shown to induce resistance to the israeli acute paralysis virus (IAPV)^12^, and even large scale application of dsRNA against this virus has been attempted^13^. Also in mosquitos such as the *Aedes aegypti*, a vector for many human diseases like the zika and dengue virus, trials have been performed. Genetically manipulated *A*. *aegypti* expressing inverted-repeat dengue virus RNA sequences showed an increased protection against the virus^14,15^, and recently it was shown that RNAi can be induced by soaking larvae in a dsRNA solution^16^, important for using the method as a viral control measure.

Salmon louse (*Lepeophtheirus salmonis* Krøyer, 1838) is a marine ectoparasitic copepod on salmonid fish, representing a severe problem for the salmon farming industry due to antiparasitic treatment resistance and the consecutive impact on wild salmonid fish^17–20^. Its life cycle consists of eight developmental stages each separated by a molt^21,22^. The two initial instars, nauplius I and II, are planktonic, whereas the next instar, the copepodid, detects and attaches to the epidermis or gill of the host fish. Here the louse passes through two chalimus and two pre-adult stages before the final molt to adult. The louse feeds on mucus, skin, and blood^23,24^; hence, lice infestation can increase the susceptibility to other pathogens as it disturbs the osmotic balance and stress the fish^25–27^. Thereby, RNAi has been used to study the function of various salmon louse genes by soaking of nauplius I larva or by injections of the larger pre-adult and adult stages^28–32^, in order to identify potential targets for new treatments and countermeasures.

Recently, two vertically transmitted salmon louse rhabdoviruses, LsRV-No9 and No127, have been identified^33^. Rhabdoviruses are a family of enveloped viruses with a non-segmented negative-sense single-stranded RNA genome, infecting a variety of host such as mammals, fish, birds, reptiles, insects, crustaceans and plants^34^. The LsRVs have been identified in salmon louse gland tissue^33^, and is potentially secreted onto the salmonid host skin where the expression of genes generally believed to be virus induced often are found to be moderately increased following lice infestation^35–37^. Studies on transmission routes and how the LsRVs affect lice biology and the ability to immune modulate the host is therefore of importance. There are at present no cell lines or infection models available for propagating the LsRVs^33^. In order to study LsRV transmission routes and their effect on louse biology and the host-parasitic interaction, we therefore aimed to produce virus infected (LsV) and virus free (LsVF) salmon louse strains from a common virus infected origin by using RNA interference. Prior to this, the presence of the LsRVs in lice from or near Norwegian salmon farms was analysed, as to be able to make strains reflecting the wild population of salmon lice.

## Results and Discussion

### Prevalence of the LsRVs

**Figure 1:**
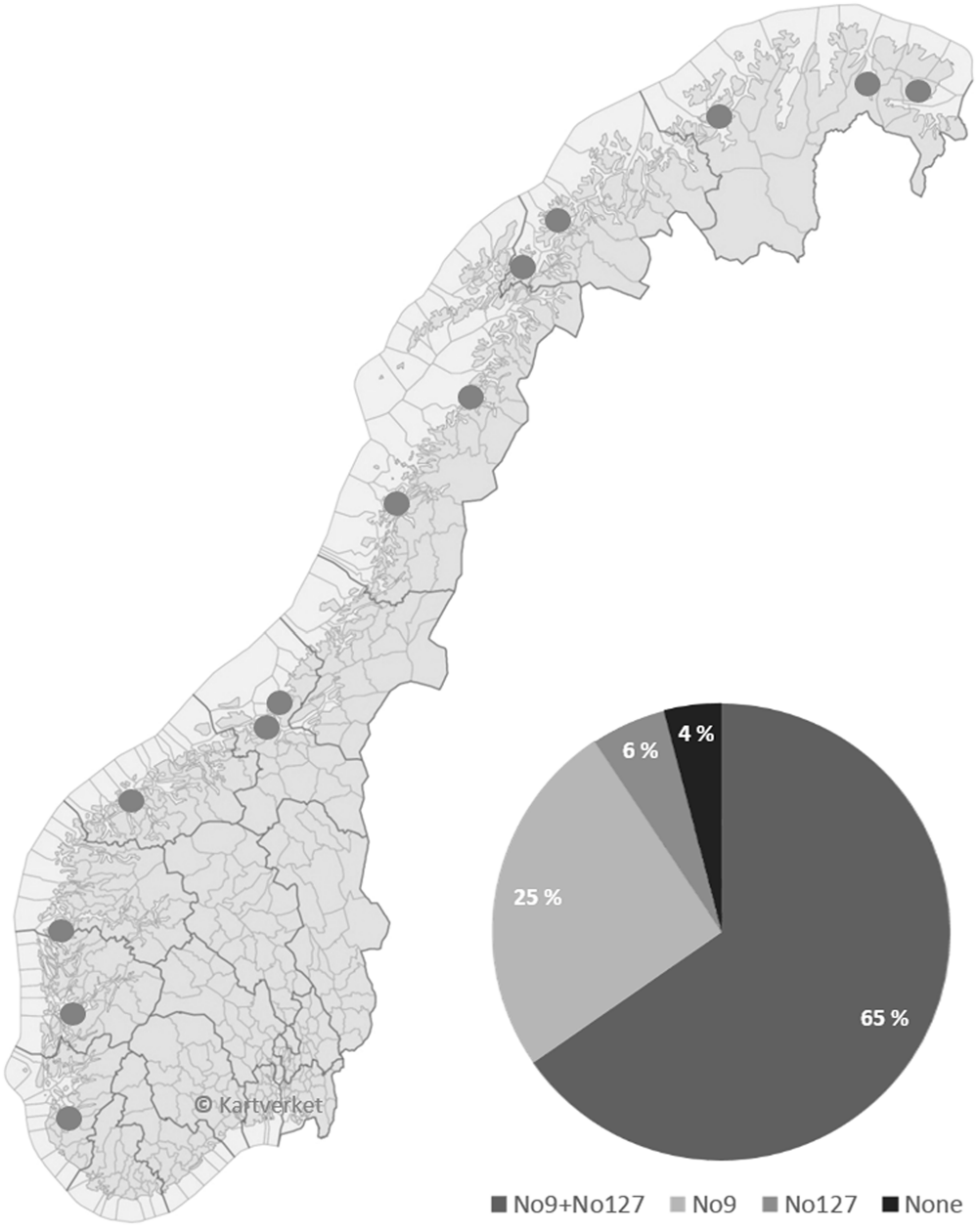
Prevalence of the two LsRV strains No9 and No127 in 75 lice sampled from farmed fish or fish nearby farms at 13 locations along the Norwegian cost. Samples were obtained from: Veranger, Tana, Alta, Senja, Harstad, Bodø, the coast of Helgeland, Bjugn, Agdenes, Romsdalen, Gulen, Hardanger and Dirdal (gray circles, location names listed from North to South). The graph shows the frequency of lice with both viral strains (No9 and No127), with No9 only, with No127 only, and with none of the two viral strains. The map, “Kommuner med hav”, was downloaded from the Norwegian Mapping Authority (https://kartkatalog.geonorge.no/metadata/kartverket/norge-illustrasjonskart/a374f867-60c0-4524-9eda-b15ab4d12858), and incorporated into the figure after changing it to grayscale and excluding text. License of use: https://creativecommons.org/licenses/by/4.0/deed.no.

The presence of LsRV-No9 and No127 in salmon louse sampled from farmed and wild hosts was examined along the Norwegian coast in order to identify the distribution and co-occurrence of these viruses. Both viruses were present at all locations tested, and most lice carried both viruses (65%). Single infections of LsRV-No9 was observed in 25% of the lice, while 6% was positive for the No127 strain only (Fig. 1), giving an overall prevalence of 90 and 71% for the No9 and No127 strains, respectively. This indicates that the LsRVs are omnipresent at sites with extensive farming of Atlantic salmon. Our laboratory strains of salmon louse were further analyzed for the presence of the two LsRVs, however, only the LsRV-No9 was detected in the LsGulen and LsAlta strains, while the LsOslo strain was negative for both viruses.

### Specificity of knockdown

**Figure 2:**
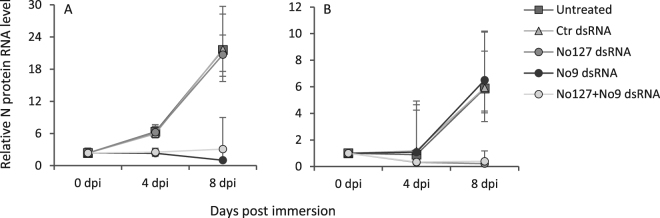
Average relative expression ± SD (N = 3) of the viral (**A**) LsRV-No9 and (**B**) No127 N protein RNA in untreated group, control group immersed with a cod CPY dsRNA, No9 group immersed with No9 N protein dsRNA, No127 group immersed with No127 N protein dsRNA, and in No9&No127 group immersed with both N protein dsRNAs.

RNA interference represents an important sequence-specific innate immune response towards viruses in invertebrates^38,39^, however, sequence-independent responses have been reported in crustaceans like shrimps^40,41^. Therefore, the specificity of viral knockdown was analyzed in the present study. The viral N protein RNA level decreased in free-living copepodids soaked in LsRV N protein dsRNA and not in lice soaked in control dsRNA or N protein dsRNA targeting the other rhabdovirus strain (Fig. 2). Hence, the induced anti-viral response was considered sequence-specific. An increase in the viral N protein RNA level were seen in control animals at 8 dpi, indicating that the LsRV-No9 overcomes the salmon louse RNAi defense mechanism likely as the viral dsRNA formed during viral replication and transcription are concealed from the RNAi system. In other rhabdoviruses, encapsulation of the viral genome during replication and the use of the ribonucleoprotein complex as template for primary transcription rather than the naked RNA have been demonstrated^34,42^.

### Viral knockdown and production of a virus free lice strain

**Figure 3:**
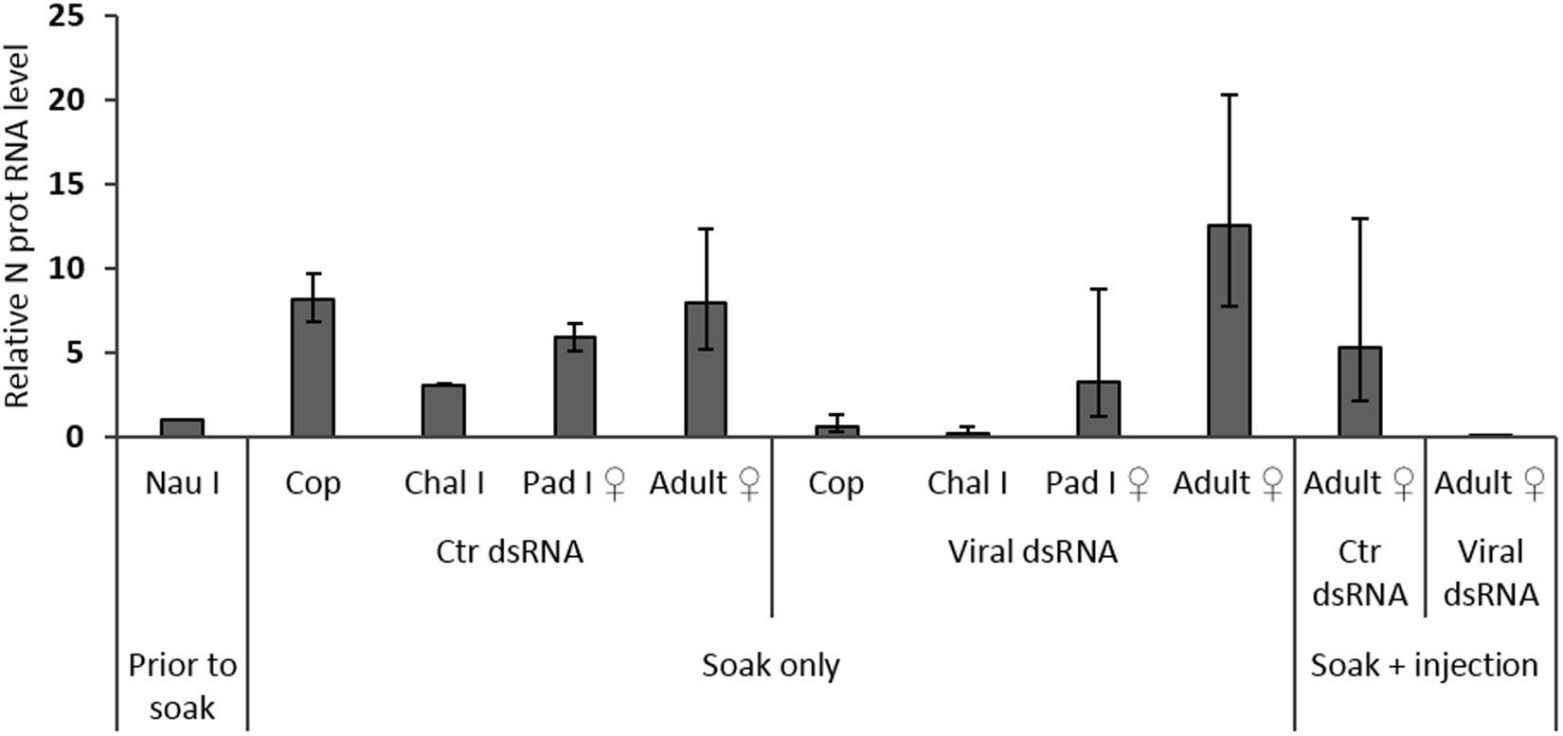
Relative N protein level (2^−ΔΔCt^) ± SD (N = 5) in control and knock-down F0 animals (exp 1) related to the viral RNA level in Nauplius I larva (nau I) prior to soaking. Cop – copepodids, chal I – chalami I, pad I – pre-adult I.

After confirming the specificity of viral knockdown, attempts to produce virus free lice strains using the LsRV-No9 infected LsGulen strain were made. Samples obtained from the LsVF_1_ F0 generation of lice at the chalimus I, pre-adult and adult stages displayed a gradual increase in viral N protein RNA level after the first pre-adult stage (LsVF_1a_, Table 1, Fig. 3), suggesting that only one RNAi treatment is not sufficient to produce virus free egg bearing adults. Also in previous RNAi studies targeting salmon louse genes, a decrease in knockdown during development has been demonstrated^43^. Therefore, a group of lice were given a second dsRNA treatment at the pre-adult stage, and an average knockdown of 97 and 87% was achieved in adult males and females, respectively (LsVF_1b_, Table 1). Viral N protein RNA were not detected in six out of ten adult female lice sampled or in the following LsVF_1b_ generations. These results were confirmed in experiment 2, using a lower density of knockdown lice on the host fish. Here, an average knockdown of 99.9% was seen and six out of eight female lice from the dsRNA treated group were LsRV negative (Table. 1). N protein RNA was not detected in the offspring from these eight lice, including the offspring from two slightly positive females. Probably, a certain level of LsRV-No9 virions needs to be present in the parenting female lice to ensure vertical transfer of the virus. Moreover, no viral N protein RNA was detected in subsequent generations. A second round of dsRNA treatment at the pre-adult stage thus increases the percentage of negative lice substantially, and is therefore required to establish a virus free strain of salmon lice. It was not tested whether an injection at the pre-adult stage only is sufficient to produce virus-free offspring, however; at this point in development the ovaries have started to develop^44^, increasing the risk of transferring the LsRVs during reproduction.

**Table 1:** RNAi mediated down regulation of the viral N protein genes in treated *L*. *salmonis*.

Exp	Lice origin	Virus strain	Generation	Lice strain	Treatment	DPI	Stage	% down regulation	Neg/sampled	Neg offspring	N generations
1	LsGulen	No9	F0	LsVF_1a_	Soak	0	cop free	91 ± 5.8			
						8	chal I	92.3 ± 7.5			
						25	pad I ♂	42.4 ± 50.6	2/5		
						25	pad II ♀	55.7 ± 43.6	1/5		
						55	adult ♀	40.9 ± 54	1/5		1
				LsVF_1b_	Soak + inject	55	adult ♂	87.3 ± 17.6	3/5		
						55	adult ♀	97.1 ± 4.9	6/10	3/5	3
2	LsGulen	No9	F0	LsVF_2_	Soak + inject	55	adult ♀	99.9 ± 0.03	6/8	8/8	4
3	LsHardanger	No9/No127	F0	LsVF_3_	Soak + inject	75	adult ♂	95.3 ± 1.8/94.1 ± 2.2	0/5		
						75	adult ♀	66.5 ± 47.9/83.2 ± 35.9	0/9	1/5	1
4	LsHardanger	No9/No127	F0	LsVF4_a_	Soak + inject	55	adult ♀	98.6 ± 2.4/97.4 ± 2.7	0/13	3/5	1
			F1	LsVF4_b_	Soak + inject	55	adult ♀	100 ± 0/100 ± 0	11/11	5/5	4

Data from experiment 1–4 are listed, showing the lice origin, virus strain, percent down regulation at various days post infection (DPI) and the number of virus negative lice. Percent down-regulation ± SD were calculated from pooled samples of planktonic copepodids (cop free) and chalimus I (chal I), and from single animals for the pre-adult (pad) and adult stages. LsV = virus infected strain, LsVF = virus free strain All the subsequent LsVF generations maintained post treatment were tested (N generations) for presence of virus and found negative.

**Figure 4:**
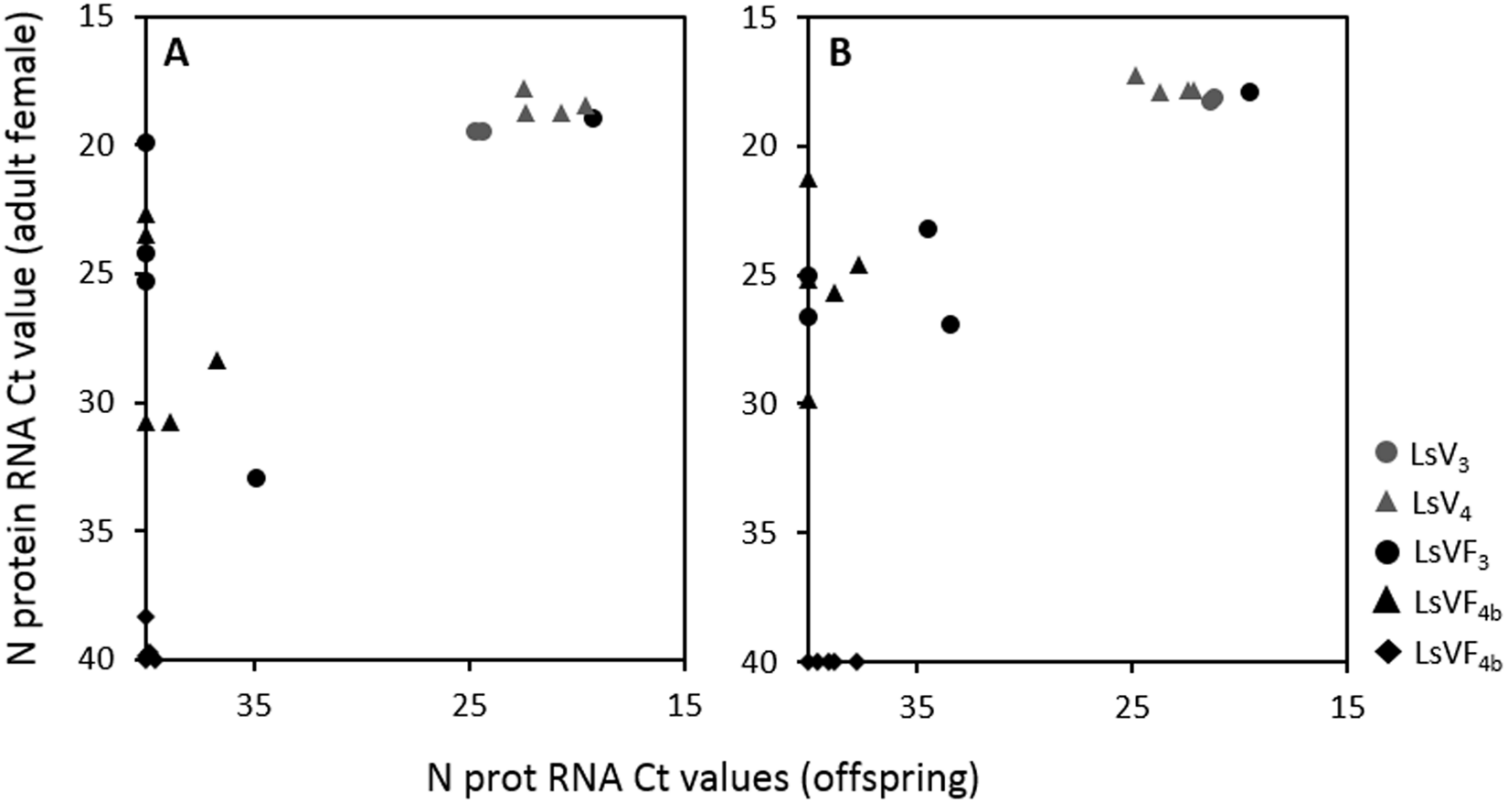
N protein RNA Ct values in parenting females LsV_3_ and LsVF_3_ (75 days post infestation (dpi)) and LsV_4_, LsVF_4a_ and LsVF_4b_ (56 dpi) plotted against the Ct values of their respective offspring at the copepodid stage. (**A**) LsRV-No9 and (**B**) No127. The reference gene was highly stable among the samples. A Ct value of 40 indicates undetected levels of viral RNA.

To test if it was possible to knockdown both LsRVs simultaneously, lice from the Hardanger fjord (LsHardanger) were taken into the lab and confirmed to be positive for both LsRVs. Eggs were incubated and lice were subjected to dsRNA treatment as in previous experiments. However, in this third experiment the LsV_3_ and LsVF_3_ F1 generations originated from the third egg string pair of adult F0 females, displaying a modest average knockdown of 66 and 83% of LsRV-No9 and No127 strain, respectively (Table 1). Thereby, only one out of five females produced offspring negative for both viruses (Fig. 4). RNAi efficiency has been shown to decline when attempting to knockdown two salmon louse genes simultaneously^45^. However, as demonstrated in experiment 1, viral RNA level rise over time post dsRNA treatment, and when using the first egg string pairs in experiment 4a, three out of five F0 females produced negative offspring (LsVF_4a_). This knockdown success is not altogether very different from that in experiment 1 and 2. This demonstrates the importance of collecting early egg strings to establish the virus free F1 generation, before the viral level increases above the threshold for vertical transmission. This also demonstrates that it is possible to simultaneously knockdown both viruses sufficiently to produce virus free strains from a double infected origin. Interestingly, in experiment 4b, dsRNA treatment of the nauplius cohort originating from LsVF_3_ F0 females apparently cured all treated individuals, and the LsVF_4b_ strain remained negative for three generations (Table 1). This nauplius cohort had lower virus levels than normal due to the dsRNA treatment in the previous generation, and this may explain the high treatment efficiency. This suggests that increasing the dose of administered dsRNA may potentially remove both viruses from a lice cohort over one generation. However, since is it not possible to analyze the viral status of lice without sacrificing them, it is recommended to use the F1 generation to obtain material for experimental studies rather than curing the F0 generation.

### Horizontal transmission of LsRV-No9

**Figure 5:**
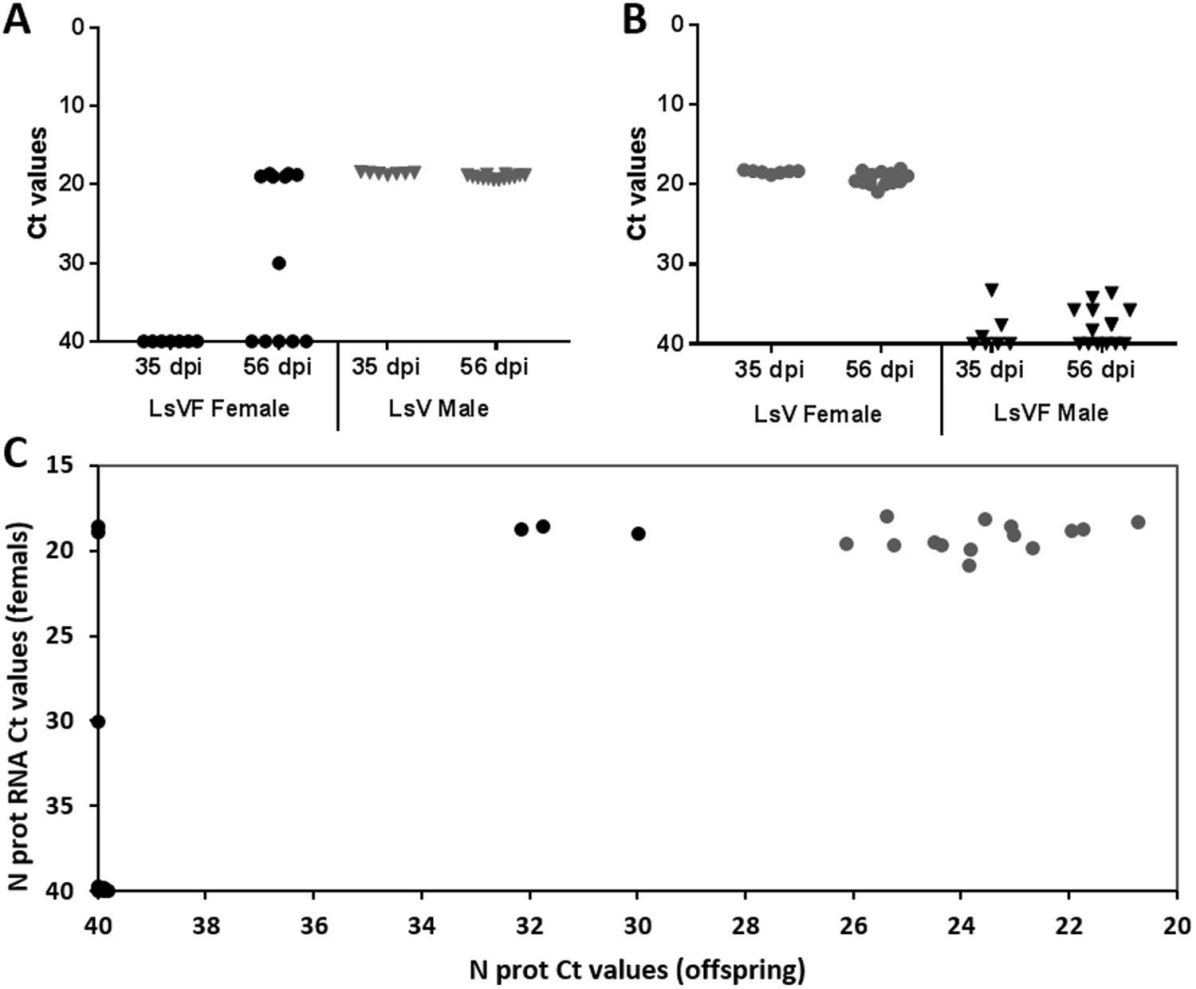
Level of LsRV-No9 N protein RNA given as average Ct values for each biological replicate in the transmission experiment. (**A**) LsVF_2_ females (black circle) cohabitated with LsV_2_ males (gray triangle). (**B**) LsV_2_ females (gray circle) cohabitated with LsVF_2_ males (black triangle). (**C**) Ct values from LsV_2_ and LsVF_2_ females sampled at 56 days post infestation (dpi) plotted against the Ct values of their respective offspring at the copepodid stage. A Ct value of 40 indicates undetected levels of viral RNA. The reference gene was highly stable among the samples.

The degree of horizontal transmission of the LsRV-No9 virus was studied due to its wide distribution along the Norwegian coast. Virus free adult males (LsVF_2_) were cohabitated with virus infected pre-adult females (LsV_2_) on naïve fish and later sampled as adults. After 35 days of cohabitation, three out of seven males had become marginally positive for the LsRV-No9 and eight out of 14 lice slightly positive at 56 days of cohabitation (Fig. 5a). As the LsRVs have been localized to lice glandular tissue and on salmon skin at the chalimi attachment site^33^, it is likely that virus particles are secreted onto the salmon skin and potentially consumed by the lice. The uptake of virus particles through the gut seems, however, to be limited as the level of viral RNA was low, still after 56 days of cohabitation. This could suggest that virus particles were only present in the gut content of the positive males or adhered to the louse mucoid coat. Thus starving LsVF male cohabitants in continuous flow-through incubators prior to analysis could answer whether males obtained the infection horizontally.

To study transmission of LsRV-No9 from males to females, pre-adult II LsVF_2_ females were placed on naïve fish together with LsV_2_ adult males. After 35 days of cohabitation the females had molted into adults, become fertilized by LsRV-No9 positive males by the attachment of a sperm sac, and produced their first external fertilized egg strings. At this stage none of the females were LsRV positive (Fig. 5b), hence if viral particles were transferred with the spermatophore, the viral RNA level must have been below the detection limit. After 56 days of cohabitation, seven out of 13 LsVF_2_ females were positive, and six of these had equally high viral N protein RNA level as the LsV_2_ lice. Surprisingly, four of these seven positive LsVF_2_ females produced negative offspring, while the other three females produced only marginally positive offspring (Fig. 5c). In contrast, the LsV_2_ females, with similar N protein RNA level, all produce virus infected offspring displaying significantly higher N protein RNA levels. Presumably, the virus was vertically transmitted from females horizontally infected by males, and not directly from male sperm. The females were infected by virus particles entering either through the gut, or via the seminal fluid delivered during mating. Considering that LsVF_2_ males showed only marginally elevated levels of viral RNA after cohabitation with LsV_2_ females, the latter is more likely. Similarly, sigma viruses that infect Mediterranean fruit flies (*Ceratitis capitata*), *Drosophila immigrans*, and speckled wood butterflies (*Pararge aegeria*) have been shown to mainly transmit maternally, with only lower rates of paternal transmission seen^46^. The presence of highly positive females producing offspring that were either negative or slightly positive for the LsRVs indicates that the infection needs to develop beyond a threshold for vertical transfer in females before entering the eggs at some point during oocyte development. At any point in time, an adult female carry at least four batches of eggs in different phases of development. One batch are developing embryos within external egg strings, one batch of eggs undergoing vitelogenesis in the genital segment, one batch developing in the oviduct (this phase include the development of oogonium cell walls) and at least one batch of future oocytes developing within the large tubular syncytium that make up the ovary^44,47^. At 10 °C, each batch require around 9–10 days before moving to the next stage of development^48^. Thus, the oocytes giving rise to the egg strings collected at 56 days were formed in the ovary roughly 20–30 days earlier, at a point in time where females tested negative for the virus. Previously, high levels of both LsRV transcripts have been detected in the salmon louse ovaries^33^. Altogether, this suggests that the LsRV-No9 virus transmits to eggs in an early phase of oocyte development.

### Conclusion

The present study shows that it is possible to simultaneously remove two vertically transmitted rhabdoviruses from a strain of *L*. *salmonis* by subjecting the lice to two dsRNA treatments during development. As the degree of horizontal transmission of LsRV-No9 through the salmon louse gut seems to be limited, the main mode of transmission is most likely vertically as previously suggested^33^. A low horizontal transfer rate may have been vital for the present success of RNAi mediated viral treatment in salmon louse since variability in the degree of knock-down and virus proliferation could potentially have produced a significant horizontal infection pressure increasing the virus level above the threshold for vertical transfer. The present study indicates that LsRV-No9 is, although to a lesser extent, horizontally transmitted with male sperm. This emphasizes the need to sample the first egg string before the females become re-infected by virus positive males. The fact that multiple infections appear to be common advocates the need to study transmission mechanisms in lice strains carrying several viruses. Moreover, since the LsRVs seem to be omnipresent at sites with extensive farming of salmon, further research on how these rhabdoviruses influence louse biology and the interaction between the lice and its salmonid host are of importance. As no cell lines for LsRVs cultivation are presently known, the method reported herein provide a vital fundament for further research on vertically transmitted RNA viruses in sea lice. In principle, this study also demonstrates in more general terms that it is possible to remove vertically transmitted virus from strains of arthropods by means of dsRNA treatment. This allows for experimental designs comparing virus infected and virus free strains from a common origin, reducing the genetic variability within the system observed.

## Method

### Source and culture of salmon lice

Laboratory strains of salmon lice were maintained on farmed Atlantic salmon (*Salmo salar*) according to Hamre *et al*.^49^. The salmon were hand fed on a commercial diet, and reared in sea water with a salinity of 34.5 g/kg and a temperature of 10 °C. Eggs, nauplii and copepodids were kept in seawater from the same supply. Nauplii were obtained from hatching eggs, and kept in single wells in a flow through system^49^.

Three laboratory strains were analyzed for LsRVs, originating from Gulen (LsGulen), Alta (LsAlta) and the fjord of Oslo (LsOslo). Moreover, two to ten lice taken from or near salmon farms in Varanger, Tana, Alta, Senja (Laksfjord), Bodø (Skjerstadfjorden), the coast of Helgeland, Bjugn, Agdenes, Romsdalen, Gulen, Hardanger, and Dirdal (Høgsfjorden) were analyzed for the presence of the two LsRVs.

All experimental procedures were performed in accordance with national legislation for animal welfare, and approved by the governmental Norwegian Animal Research Authority (NARA, http://www.fdu.no/fdu/).

### RNA purification and cDNA synthesis

Lice for RNA purification were stored in RNAlater (Qiagen). Total RNA from nauplius, copepodids and chalimus stages was isolated with a combined Tri reagent (Sigma Aldrich) and RNeasy (Qiagen) method, as previously described^50^. Pre-adult and adult lice were purified with Tri reagent (Sigma Aldrich) according to the Trizol reagent protocol described by Invitrogen. Samples were either frozen at −80 °C until use, or cDNA synthesis was done directly. For real time RT-PCR, cDNA synthesis was carried out using the AffinityScript qPCR cDNA Synthesis Kit (Stratagene) according to the supplier recommendations, adding 200 ng total RNA. The cDNA samples were diluted 3 times and stored at −20 °C until use. For PCR, the qScript cDNA SuperMix (Quanta Bioscience) was used, applying 1 µg total RNA.

### Virus detection

For the detection of viral N protein RNA, real time RT-PCR was performed with 1x SYBR Select Master mix (Life Technologies), 500 nM gene specific primers (Table 2) and 2 µl cDNA in 10 µl reactions. Samples were run in duplicate on an Applied Biosystems 7500 Real-Time PCR System under standard conditions (50 °C for 2 min, 95 °C for 2 min, 40 cycles of 95 °C for 15 seconds and 60 °C for 1 min, followed by a melt curve analysis at 60–95 °C). The efficiency of each assay was tested with a five-point standard curve of 4-fold dilutions, given by the equation E% = (10^1/slope^ − 1) × 100 ^51^. The salmon louse elongation factor 1 alfa (eEF1α) was used to normalize the data^52^, and always included in the run. When the relative differences in threshold cycle between the viral genes and the reference gene (ΔCT) and expression relative to controls (ΔΔCT) were calculated, they were transformed by the equation 2^−ΔΔCT^ ^53^. If only CT values were displayed, the reference gene were always run and evaluated to be stably expressed. For detection of viral RNA in pre-adult and adult animals, one animal per sample was purified, while 10–50 animals were pooled in one sample for the copepodid and chalimus stages.

**Table 2:** Primers used for virus detection and production of dsRNA.

Name	Primer 5′ → 3'	Application
Fw1 LsRV-No9	TCCAGTTAGAAGACGGCTTGATCGGACG	Virus detection
Rev1 LsRV-No9	CACCATGCTACAGCTTCCCTGGGAGTC	Virus detection
Fw1 LsRV-No127	CACCAGCCAGTTTCCCGTCTCAATGG	Virus detection
Rev1 LsRV-No127	CGACGGGGTTCCAGGTTATATCGGACA	Virus detection
Fw2 LsRV-No9	TTCTCCCGAACCGACATGGA	RNAi
Rev2 LsRV-No9	AGGGGATTGGCGGTGACTGA	RNAi
Fw2 LsRV-No127	GGAGCCATCGGAGGTTATGACC	RNAi
Rev2 LsRV-No127	AAGGGGCCGTGTCAATCCTA	RNAi

### RNA interference

RNAi was performed as previously described^28,30^, with primers listed in Table 2. In short, the MEGAscript RNAi Kit (Ambion) was used to produce double stranded RNA according to supplier’s instructions. The dsRNA was made targeting the N protein of the two viruses (Accesion no.: KJ958535 and KJ958536). For soaking, a batch of newly hatched nauplii was incubated overnight in 1.5 µg of each dsRNA. After molting into the nauplius II stage, the nauplii were returned to flow through incubators. RNAi in pre-adults was performed by injecting around 250 ng of each dsRNA into the cephalothorax.

### Specificity

To analyze the specificity of the knock-down, nauplius I from three females positive for both viruses were each divided into five groups. One group were not added any dsRNA (untreated) while the rest of the groups were added control dsRNA, N protein dsRNA from the No9 strain, N protein dsRNA from the No127 strain, or N protein dsRNA from both No9 and No127 strain. Samples were taken at 0, 4 and 8 days after soaking for analyzing the viral N protein RNA level.

### Establishment of virus free sea louse strains using RNA interference

**Figure 6:**
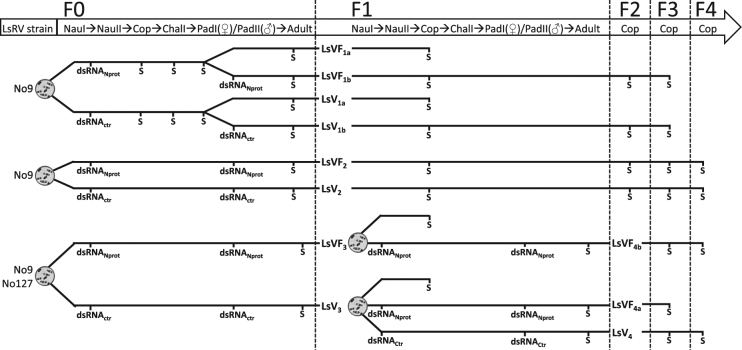
Experimental setup and sampling regime in the four experiments (Exp 1–4) aiming to establish virus free lice strains (LsVF_1_-LsVF_4_) and their corresponding virus infected strains of common origin (LsV_1_-LsV_4_). Parental generation is denoted F0, and subsequent generations are F1-F4. Sampling points are marked with an S and show which stages are sampled and tested for presence or viral RNA in each generation/strain. NauI = nauplius 1, NauII = nauplius 2, Cop = planktonic copepodid, ChalI = chalimus 1 stage, Pad = preadult. The timing for administration of double stranded RNA (dsRNA) is also given, either by soaking at the Nauplius I to Nauplius II transition, or by injection at the pre-adult I (females)/pre-adult II (males) stage. The dsRNA was either a mixture of N protein RNA from both LsRV-No9 and –No127 strains (dsRNA_Nprot_), or control dsRNA (dsRNA_ctr_).

The general procedure applied for establishing a virus free strain of salmon louse involved RNAi knock-down of the LsRV N protein to inhibit virus replication in a F0 generation of adult females in order to inhibit vertical transmission and produce a virus free F1 generation. To produce a virus free and virus infected strain, naïve fish kept in single tanks were infested with around 60–80 LsRV N protein knock-down copepodids or untreated copepodids. When a second round of RNAi was administered, pre-adult I females and pre-adult II males were removed from the fish and injected with dsRNA before they were returned to naïve fish and allowed to develop into adults. Thereafter lice were sampled and their egg strings hatched in individual incubators^49^. Each female and her offspring were tested for presence of virus to select the F0 parents for the virus free strain (LsVF), parents for the virus infected strain (LsV) were selected from the control fragment group. Altogether, four experiments were carried out, and strains established in each of the experiments were named according to the experiment number (n), LsVF_n_ and LsV_n_ (Fig. 6).

Experiment 1: LsVF_1_ and LsV_1_ F0 were established from the LsGulen strain (LsRV-No9 positive) and hosted on 6 fish/strain. One fish from each group were sampled at 8 days post infestation (dpi), 25 dpi and 55 dpi (LsVF_1a_). To test whether an additional RNAi treatment at the pre-adult stage can improve the LsRV N protein knock-down in adults, a group of LsVF_1_ named LsVF_1b_ were established by administering a second round of RNAi to 15 female and 12 male pre-adult lice, and putting them on naïve fish (n = 3). Surviving lice were sampled again at the adult stage (55 dpi) and their egg strings were placed in separate incubators. Copepodids were checked for presence of virus, and the F1 generation was founded based on the offspring from one F0 virus negative female and followed for two more generations.

Experiment 2: LsV_2_ and LsVF_2_ were established from the LsGulen strain and the F0 generation was hosted on 3 fish/strain. At 26 dpi, all lice were sampled and given a second round of RNAi. Subsequently, for each of the two strains, four naïve fish were infested with ten F0 females and seven F0 males and allowed to develop into adults. At 58 dpi, the lice were sampled and the first egg string collected. The F1 copepodids were analyzed for the presence of viral N protein. The LsV_2_ and LsVF_2_ strains were further propagated for three generations.

Experiment 3: LsVF_3_ and LsV_3_ were based on lice from the Hardanger fjord hosting both the LsRV-No9 and No127 virus strains. In this experiment both viruses were knocked down simultaneously to create the LsVF_3_ strain. The F0 generation of each strain was hosted on three fish respectively, and following dsRNA injection at the pre-adult I stage, ten female and male lice were put back on 3 fish/strain, and then sampled at 75 dpi when the lice carried their third egg string.

Experiment 4: Since the F1 generation of LsVF_3_ was slightly positive, two new virus free strains were made: LsVF_4a_ based on LsV_3_, and LsVF_4b_ based on LsVF_3_. Also note that the LsV_4_ F0 was based on the LsV_3_ F1 generation. For all three strains, ten female and eight male lice per fish were added to three fish after a second RNAi treatment at the pre-adult stage. Further, the first egg string produced by the females was sampled at 55 dpi, and the LsVF_4b_ strain was followed  for three generations.

### Transmission experiment

To study the degree of horizontal transmission of LsRV-No9, pre-adult II females and adult males from the F2 generation of LsV_2_ and LsVF_2_ were used to infest two groups of naïve fish. Ten LsV_2_ female lice were put together with ten LsVF_2_ males on one fish in a single fish tank, while ten LsVF_2_ female lice were put on a second fish together with ten LsV_2_ males. After 35 days, when the females had become egg bearing adults (63 dpi), the lice were sampled and tested for virus.

A second transmission experiment was carried out using the LsV_2_ and LsVF_2_ F3 generation, with an equal experimental design, only this time adding eight females and eight males to each of three fish for each combination. Adult lice were sampled after 56 days (81 dpi), and the level of viral RNA was analyzed. Egg strings were collected and incubated in flow-trough incubators, and the copepodids were sampled and tested for virus by real time RT-PCR.

The datasets generated during and/or analyzed during the current study are available from the corresponding author on reasonable request.

We carefully reviewed the ethical standards of the journal and we hereby certify that the procedures used with the investigated species comply fully with those standards.
